# Evaluation of Dynamic Contrast‐Enhanced MRI Measures of Lung Congestion and Endothelial Permeability in Heart Failure: A Prospective Method Validation Study

**DOI:** 10.1002/jmri.28174

**Published:** 2022-03-27

**Authors:** Joseph Cheriyan, Alexandra Roberts, Caleb Roberts, Martin J. Graves, Ilse Patterson, Rhys A. Slough, Rosemary Schroyer, Disala Fernando, Subramanya Kumar, Sarah Lee, Geoffrey J.M. Parker, Lea Sarov‐Blat, Carmel McEniery, Jessica Middlemiss, Dennis Sprecher, Robert L. Janiczek

**Affiliations:** ^1^ Research GSK Clinical Unit Cambridge Cambridge UK; ^2^ Division of Experimental Medicine and Immunotherapeutics University of Cambridge Cambridge UK; ^3^ Cardiovascular Clinical Trials Office Cambridge University Hospitals NHS Foundation Trust Cambridge UK; ^4^ Clinical Imaging GSK Stevenage UK; ^5^ Imaging Services Bioxydyn Ltd Manchester UK; ^6^ Department of Radiology University of Cambridge Cambridge UK; ^7^ Statistics GSK Collegeville Pennsylvania USA; ^8^ Consulting Amallis Consulting Ltd London UK; ^9^ Centre for Medical Imaging Computing, Department of Computer Science University College London London UK; ^10^ Research and Development GSK, Crescent Drive Philadelphia Pennsylvania USA; ^11^ Consulting BioView Consulting LLC Blue Bell Pennsylvania USA

**Keywords:** DCE‐MRI, edema, heart failure, pulmonary interstitium, lung congestion

## Abstract

**Background:**

Methods for accurate quantification of lung fluid in heart failure (HF) are needed. Dynamic contrast‐enhanced (DCE)‐MRI may be an appropriate modality.

**Purpose:**

DCE‐MRI evaluation of fraction of fluid volume in the interstitial lung space (*v*_*e*_) and vascular permeability (*K* ^trans^).

**Study Type:**

Prospective, single‐center method validation.

**Population:**

Seventeen evaluable healthy volunteers (HVs), 12 participants with HF, and 3 with acute decompensated HF (ADHF).

**Field Strength/Sequence:**

*T*_1_ mapping (spoiled gradient echo variable flip angle acquisition) followed by dynamic series (three‐dimensional spoiled gradient‐recalled echo acquisitions [constant echo time, repetition time, and flip angle at 1.5 T]).

**Assessment:**

Three whole‐chest scans were acquired: baseline (Session 1), 1‐week later (Session 2), following exercise (Session 3). Extended Tofts model quantified *v*_*e*_ and *K* ^trans^ (voxel‐wise basis); total lung median measures were extracted and fitted via repeat measure analysis of variance (ANOVA) model. Patient tolerability of the scanning protocol was assessed.

**Statistical Tests:**

This was constructed as an experimental medicine study. Primary endpoints: *K* ^trans^ and *v*_*e*_ at baseline (HV vs. HF), change in *K* ^trans^ and *v*_*e*_ following exercise, and following lung congestion resolution (ADHF). *K* ^trans^ and *v*_*e*_ were fitted separately using ANOVA. Secondary endpoint: repeatability, that is, within‐participant variability in *v*_*e*_ and *K* ^trans^ between sessions (coefficient of variation estimated via mixed effects model).

**Results:**

There was no significant difference in mean *K* ^trans^ between HF and HV (*P* ≤ 0.17): 0.2216 minutes^−1^ and 0.2353 minutes^−1^ (Session 1), 0.2044 minutes^−1^ and 0.2567 minutes^−1^ (Session 2), 0.1841 minutes^−1^ and 0.2108 minutes^−1^ (Session 3), respectively. *v*_*e*_ was greater in the HF group (all scans, *P* ≤ 0.02). Results were repeatable between Sessions 1 and 2; mean values for HF and HV were 0.4946 and 0.3346 (Session 1), 0.4353 and 0.3205 (Session 2), respectively. There was minimal difference in *K* ^trans^ or *v*_*e*_ between scans for participants with ADHF (small population precluded significance testing). Scanning was well tolerated.

**Data Conclusion:**

While no differences were detected in *K* ^trans^, *v*_*e*_ was greater in chronic HF patients vs. HV, augmented beyond plasma and intracellular volume. DCE‐MRI is a valuable diagnostic and physiologic tool to evaluate changes in fluid volume in the interstitial lung space associated with symptomatic HF.

**Level of Evidence:**

2

**Technical Efficacy Stage:**

2

Lung congestion (or lung edema), defined as fluid accumulation in the interstitial and often alveolar space of the lungs, is common in patients with heart failure (HF).^1^ As this clinical setting has considerable morbidity and mortality implications, understanding its pathophysiology remains a top priority. Current imaging techniques for assessing lung water include qualitative chest radiographs, ultrasound, and computed tomography scans.^2^, ^3^ While chest radiographs are generally the most readily available, their application is hampered by interobserver variability and low sensitivity.^3^ MRI has the potential to objectively quantify lung congestion and has demonstrated increased lung water content (including vascular volume) in patients with HF compared with healthy volunteers (HVs) using proton‐density‐weighted MRI.^4^, ^5^ Detecting and quantifying small fluid shift changes between various lung compartments may offer insights into the induction as well as resolution of clinically symptomatic HF and in the evaluation of mechanism or efficacy of new drugs being developed for the condition. However, quantification remains problematic due to difficulties in distinguishing between intravascular and extravascular water with currently available techniques. Use of a gadolinium‐based contrast agent combined with MRI may enable this distinction, providing the plasma volume fraction (*v*_*p*_), the interstitial volume fraction (*v*_*e*_), and the transfer constant between *v*_*p*_ and *v*_*e*_ (*K* ^trans^) (Fig. 1), which is strongly dependent on vascular permeability as well as blood flow and vascular surface area.^6^

**FIGURE 1:**
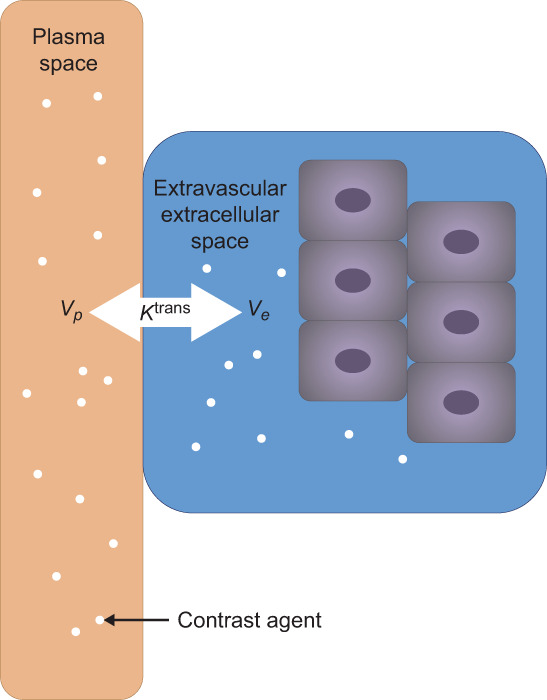
Relationship between plasma volume fraction (*v*_*p*_), the interstitial volume fraction (*v*_*e*_), and the exchange between *v*_*p*_ and *v*_*e*_ (*K* ^trans^).

Lung congestion is traditionally viewed as the result of increased hydrostatic pressure leading to fluid extravasation.^7^ This is associated with increased pulmonary vascular abnormalities due to enhanced fibrosis and matrix deposition, resulting in higher mortality rates in patients with HF.^8^ HF is classically described as a clinical entity relating primarily to a reduced left ventricular ejection fraction, the underperfusion of various organs, and occasionally hypotension. However, the primary cause of hospitalization for acute HF often does not directly relate to these manifestations, but rather to lung congestion symptoms, such as dyspnea and the ultimate onset of acute pulmonary edema.^1^ An imaging‐based method to quantify the distribution of fluid could elucidate this physiology and facilitate discovery of alternative therapeutic options.

Dynamic contrast‐enhanced (DCE)‐MRI has previously been used to detect alterations in vascular permeability and interstitial water volume in the lungs of smokers relative to HV^9^ and vascular flow and permeability in patients with lung cancer.^10^, ^11^ DCE‐MRI has also been used to assess transpulmonary circulation (pulmonary transit time) in patients with HF and HV.^12^ However, we are unaware of literature reporting DCE‐MRI use to assess lung congestion in patients with HF.

The aim of this study was to evaluate DCE‐MRI measurements of the fraction of fluid volume in the interstitial lung space (*v*_*e*_) and vascular permeability (*K* ^trans^) in HV and participants with HF or acute decompensated HF (ADHF). We aimed to assess this in different temporal and exercise settings to determine its utility in quantifying lung water between the different compartments as well as developing an experimental technique to better understand fluid hemodynamics, before and after employing licensed or more novel drug treatments.

## Materials and Methods

The study received favorable opinion from the National Research Ethics Services Committee East of England and was registered on ClinicalTrials.gov. All participants provided written informed consent. This study (NCT02135861; GlaxoSmithKline [GSK] study number 201137) was funded by GSK.

### Participants and Study Design

This was a prospective single‐center method validation study conducted between July 30, 2014 and February 22, 2017. Forty‐one participants were screened across three groups: HV, HF, and ADHF. The HV group included the participants initially recruited, followed by participants with HF who were recruited as they were identified, as well as older HV who were recruited after a prespecified interim analysis to improve age match within the confines of the protocol.

All eligible participants (HV, HF, and ADHF) were required to be at least 18 years of age with a minimum body weight of 50 kg and a body mass index of 18 kg/m^2^ to 40 kg/m^2^. Eligible patients for the HF group had to have mild/moderate HF of any etiology (New York Heart Association Class II/III).^13^ Participants with ADHF were either dyspneic at rest or with minimal activity, had to be tachypneic (≥20 breaths/minute) and/or demonstrated rales or crackles audible on auscultation, with evidence of lung congestion/edema (via chest radiograph within the last 48 hours approximately) and at least one treatment with an intravenous diuretic prior to the first DCE‐MRI scan.

Participants were excluded if they were contraindicated for MRI scanning, were pregnant, were current smokers, were positive for drug or alcohol abuse, or had an estimated creatinine clearance <60 mL/min (<40 mL/min for ADHF). Additional exclusion criteria for participants with HF were history of known primary pulmonary disease requiring current medication or other therapy, orthopnea of sufficient severity to preclude supine scanning as determined at screening, unstable angina within the prior 3 months, uncontrolled hypertension (resting systolic blood pressure > 160 mmHg or resting diastolic blood pressure > 100 mmHg), or resting hypoxia while breathing room air (oxygen saturation < 88%). Additional exclusion criteria for participants with ADHF were end‐stage HF (defined as requiring left ventricular assist devices, intra‐aortic balloon pump, or any type of mechanical support), chronic or intermittent renal support therapy (hemodialysis, ultrafiltration, or peritoneal dialysis), ongoing or planned intravenous treatment within 1 hour of MRI scan appointment, history of known primary pulmonary disease requiring current medication or other therapy, or orthopnea of sufficient severity to preclude supine scanning.

**FIGURE 2:**
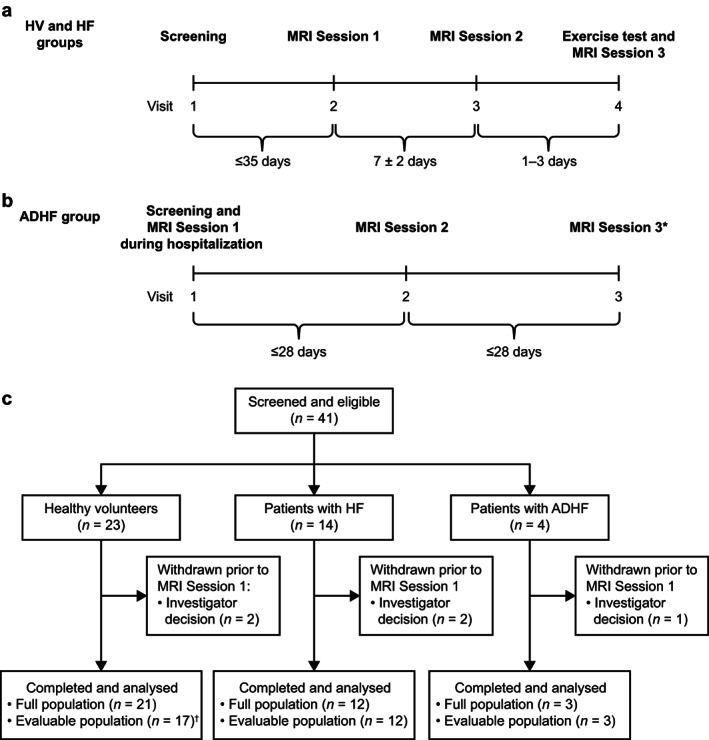
Study design for (**a**) participants with heart failure (HF) and healthy volunteers (HVs) and (**b**) participants with acute decompensated heart failure (ADHF), and (**c**) a flowchart illustrating participant inclusion/exclusion. Asterisk (*) represents that for the ADHF group, a third MRI session was required only if lung congestion was not resolved at MRI Session 2; Dagger (^†^) represents that four HV participants were excluded from the evaluable population due to age  years.

For patients with HF only, the following parameters pertaining to medical and medication history were recorded at screening: confirmation of HF class, symptoms, years since HF diagnosis, degree of exercise intolerance, presence of orthopnea and/or paroxysmal nocturnal dyspnea, peripheral edema, significant past medical history, and medication history. HV and participants with HF were screened and underwent whole‐chest DCE‐MRI imaging (MRI Session 1) and baseline procedures at least 35 days later (Fig. 2a). Repeatability (within‐participant variability) was assessed approximately 1 week later at MRI Session 2. Two bicycle exercise tests over 2 days were conducted just prior to the third imaging session on the third day of testing (MRI Session 3). Details of exercise testing are found in Supplementary Methods. After exercise, patients were taken directly to the scanner by wheelchair, which was followed by approximately 5 minutes for positioning and approximately 10 minutes until contrast injection, resulting in a gap of approximately 15 minutes between exercise and imaging. For participants with ADHF, screening and MRI Session 1 took place while the participants were hospitalized with evidence of lung congestion detected via chest radiography and were receiving standard of care intravenous diuretic treatment (Fig. 2b). MRI Session 2 was conducted once congestion was clinically resolved. A third imaging session was needed for clarity as the congestion in one participant had not resolved by the second MRI imaging session. ADHF participants did not undergo exercise testing during any of the study visits. Total exam time in the MRI scanner was recorded for each participant in the HF and HV groups. Image acquisition was performed by study radiographers, and image analysis was performed by Bioxydyn Ltd (Manchester, UK).

### Endpoints and Assessments

For the HF and HV groups, the primary endpoints were to compare *K* ^trans^ and *v*_*e*_ between the two groups at baseline (MRI Session 1) and to assess the change in *K* ^trans^ and *v*_*e*_ following the exercise test (MRI Session 2 vs. Session 3) within the groups. For the ADHF group, the primary endpoint was the change from baseline in *K* ^trans^ and *v*_*e*_ following the resolution of lung congestion (MRI Session 1 vs. Session 2/3). The secondary study endpoint was estimation of repeatability in *v*_*e*_ and *K* ^trans^ between two MRI visits approximately 1 week apart (MRI Session 1 vs. Session 2). Additional exploratory endpoints are listed in the Supplementary Methods. The requirements for supine positioning in the MRI scanner were potentially an issue for respiratory decompensation in our HF and ADHF cohorts; therefore, we recorded vitals, oxygen saturation, and electrocardiograms (ECGs) as well as any reported complications to inform future efforts (see Supplementary Methods).

Patients were stabilized by lying supine for up to 30 minutes prior to their Session 1 and 2 scans. MRI scans were performed using a 1.5‐T system (MR450, GE Healthcare, Waukesha, WI, USA) using an eight‐channel cardiac coil; Figure S1 outlines the scanning protocol. Total scan time was recorded. The DCE‐MRI protocol included variable flip angle (VFA) *T*_1_‐mapping acquisitions, using a coronal three‐dimensional radiofrequency‐spoiled fast gradient echo sequence with main parameters of repetition time/echo time = 2.03/0.83 msec; field‐of‐view (in plane) = 450 mm × 450 mm; slice thickness = 10 mm, and number of locations per slab = 18 (interpolated to 28 reconstructed slices at 5 mm); acquisition matrix = 112 × 88, flip angle = 4°/2°/7°/10° (10 repeated volumes at each flip angle). This was followed by a 7‐minute dynamic series (parameters as for *T*_1_‐mapping, flip angle = 10°, 170 repeated volumes, temporal resolution = 2.5 sec/volume). Total scan time was about 10 minutes. Additional details on the imaging protocol and image analysis are provided in the Supplementary Methods.

Respiratory gating was not used; the motion‐induced errors in model fitting were minimized by correcting data for breathing motion using nonlinear image registration. Intravenous contrast agent was injected at the start of the 15th dynamic (0.05 mL/kg; half‐dose gadolinium‐based contrast agent [Gadovist, Bayer, Berkshire, UK] administered by power injector at 1.5 mL/sec, followed by a 25‐mL saline flush). *T*_1_ relaxation time was estimated voxel‐by‐voxel by fitting the standard spoiled gradient echo signal model to the VFA data, additionally providing an estimate of the relative equilibrium signal (*S*_0_).^14^ DCE‐MRI analysis was then performed by pharmacokinetic modeling using the extended Tofts model,^15^ as directed in the Madym software tool (University of Manchester, UK; gitlab.com/manchester_qbi/manchester_qbi_public/madym_cxx/-/wikis/home), to quantify *v*_*e*_, *K* ^trans^, and *v*_*p*_. Modeling was applied on a voxel‐wise basis within a volume of interest defining both lungs in their entirety. Arterial input functions were determined in the pulmonary artery and concentrations of contrast agent were corrected for hematocrit on an individual basis, as described in Supplementary Methods. All DCE‐MRI endpoints were derived blinded to patient visit and patient status.

The 3D DCE‐MRI parametric maps in the lung were generated from the modeling output. *qS*_0_ is the MRI equilibrium signal in the lung extracted from the *T_1_* mapping process normalized to the skeletal muscle signal^16^; this was calculated voxel‐by‐voxel within each slice by normalizing the lung *S*_0_ to the *S*_0_ measured from a small, manually defined, reference skeletal muscle region of interest within the slice. An index of total extravascular extracellular water content relative to skeletal muscle, *qS*_0_ *v*_*e*_ was calculated post hoc by multiplying the 3D maps of *qS*_0_ and *v*_*e*_. Median values of *v*_*e*_, *K* ^trans^, *qS*_0_, *T*_1_ relaxation time, and *qS*_0_ *v*_*e*_ were extracted from the 3D parametric maps for total lung and lung subregions (left lung, right lung, apical left lung, basal left lung, apical right lung, and basal right lung) for subsequent statistical analysis. For *v*_*p*_, the mean value across the lung subregions was used. Segmentation of the lungs and the region of interest in skeletal muscle for *qS*_0_ measurement was performed using semiautomated methods by Bioxydyn as described in Supplementary Methods. The border between the apical and basal segments were defined as the midpoint between the lung apex and the most inferior extent of the lung in the foot‐head direction.

### Study Populations and Endpoints

The study sample size was based on feasibility and utilized results from a previous study with a similar methodology.^9^ Withdrawn HF and HV participants or those with nonevaluable DCE‐MRI data from Sessions 1 and 2 were replaced to meet the required number of participants.

Demographics, exposure to DCE‐MRI, and the DCE‐MRI endpoints were assessed in all enrolled participants who had an MRI at Session 1 and all enrolled ADHF participants who had initiated ≥1 MRI Session (hereafter referred to as the full population). Analysis of imaging measures was performed for all HF and HV participants included in the full population who were aged ≥40 years (hereafter termed the evaluable population), thus enabling comparison between HVs and HF populations of similar ages. Demographics are provided for the evaluable ADHF, HF, and HV groups; DCE‐MRI endpoints are presented for the evaluable HF and HV groups.

### Statistical Analysis

As this was a method evaluation study, an estimation approach was used for the comparisons of interest, and no formal hypothesis testing was performed. *P values* are provided for information purposes only, not for statistical inference. Although no significance level was prespecified, for descriptive purposes, a significance level of 0.05 with no adjustment for multiple comparisons has been applied to the results for hypothesis generation and to identify where additional exploration may be of interest in future research.

The primary endpoints *K* ^trans^ and *v*_*e*_ were fitted separately using analysis of variance modeling adjusting for participant population (HF or HV), visit, interaction of participant population, and visit, with participant ID as block of the repeat factor. Point estimates (eg, mean data) and associated 95% confidence intervals (CIs) were constructed to provide a plausible range of values for the mean DCE‐MRI measurements between each participant group at each session as well as the mean differences among MRI sessions within each participant group.

For the secondary endpoint, the estimation of within‐participant variability between study visits was calculated based on the mixed model. The within‐participant variability (coefficient of variation) was estimated at 100 × sqrt(exp(*MSE*) − 1), where MSE is the mean square error from the mixed effects model in *v*_*e*_ and *K* ^trans^ between MRI visits. Pearson's correlation coefficient was used to assess associations between *v*_*e*_ derived at baseline (MRI Session 2) and age of HV.

## Results

### Participant Population

Forty‐one participants were screened, among whom five were withdrawn prior to the MRI Session 1 scan at the investigators' discretion (Fig. 2c). Withdrawals due to investigator decision were due to an inability to obtain reliable scans due to habitus (*n* = 1 in the HV group; *n* = 2 in the HF group); an incidental finding in the liver requiring clinical investigation (*n* = 1 in the HV group); and a contraindication for the study procedure (*n* = 1 in the ADHF group).

Among the 36 participants in the full population, 32 underwent at least one MRI scan, were aged ≥40 years, and were therefore included in the evaluable population (Fig. 2c). Baseline demographics for the evaluable population are summarized in Table 1. Within the evaluable population, 17 were HV (mean age: 61.2 years), 12 had HF (mean age: 67.8 years), and three had ADHF (mean age: 79.3 years). The majority of participants across all three groups were male (67%–88%). Mean participant body mass index was higher in the HF group (29.2 kg/m^2^) than in the HV group (26.4 kg/m^2^) and the ADHF group (27.3 kg/m^2^). Mean values for N‐terminal pro b‐type natriuretic peptide, an important biomarker in the diagnosis of HF, were above the normal range (<400 pg/mL) for patients with both HF and ADHF. Scans were well tolerated; participants in the HF and HV groups spent a similar duration in the MRI scanner with mean examination times of 15.7 minutes (range: 12–23 minutes) and 13.8 minutes (12–19 minutes), respectively.

**TABLE 1:** Baseline Demographics

	HV	HF	ADHF
	Evaluable Population^a^ (N = 17)	Evaluable Population^a^ (N = 12)	Evaluable Population^a^ (N = 3)
Age (years)	61.2 (11.4)	67.8 (13.4)	79.3 (4.9)
Age groups, *n* (%)			
18–64 years	10 (59)	4 (33)	0
65–74 years	4 (24)	4 (33)	0
≥75 years	3 (18)	4 (33)	3 (100)
Sex, *n* (%)			
Female	2 (12)	2 (17)	1 (33)
Male	15 (88)	10 (83)	2 (67)
Weight (kg)	83.8 (13.0)	87.7 (7.7)	75.9 (5.1)
Body mass index (kg/m^2^)	26.4 (2.9)	29.2 (2.9)	27.3 (3.3)
NT‐proBNP (ng/L)	N/A	714.5 (673.8)	2757.7 (1822.5)
Ethnicity, *n* (%)			
Hispanic or Latino	0	0	0
Not Hispanic or Latino	17 (100)	12 (100)	3 (100)
Race, *n* (%)			
African American/African heritage	0	1 (8)	0
White	17 (100)	11 (92)	3 (100)

Values are reported as mean (SD) unless otherwise stated.

ADHF = acute decompensated heart failure; DCE‐MRI = dynamic contrast‐enhanced magnetic resonance imaging; HF = heart failure; HV = healthy volunteers; N/A = not applicable; NT‐proBNP = N‐terminal pro b‐type natriuretic peptide; SD = standard deviation.

^a^ Evaluable population: All HF and HV participants included in the full population^b^ aged ≥40 years; four HV participants were excluded from the evaluable population due to age <40 years. In the HF and ADHF groups, the evaluable population includes all patients in the full population.

^b^ Full population: Enrolled subjects who initiated Session 1 DCE‐MRI scan (HF and HV) or ≥1 session of DCE‐MRI or lung ultrasound scan (ADHF).

### Imaging Parameters in Participants with HF and HV

#### IMAGING MEASUREMENT REPEATABILITY AND EFFECT OF EXERCISE

**FIGURE 3:**
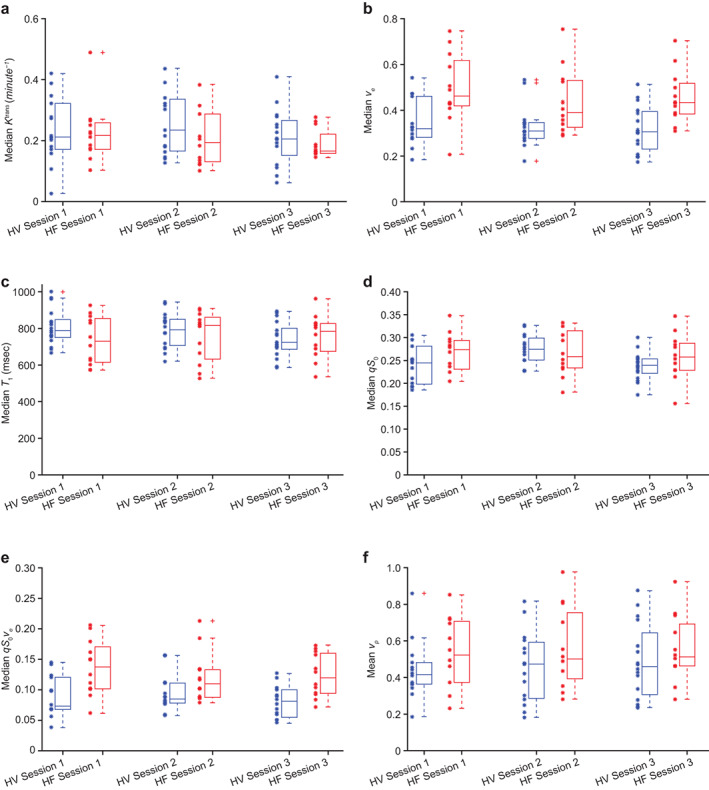
Box plot of median with individual outcome of total lung *K* ^trans^ (**a**; Session 1: *P* ≤ 0.74; Session 2: *P* ≤ 0.17; Session 3: *P* ≤ 0.37), *v*_*e*_ (**b**; Session 1: *P* ≤ 0.01; Session 2: *P* ≤ 0.02; Session 3: *P* ≤ 0.01), *T*_1_ relaxation time (**c**), *qS*_0_ (**d**), and *qS*_0_ *v*_*e*_ (**e**) and box plot of mean with individual outcome of total lung *v*_*p*_ (**f**) in participants in the evaluable populations with heart failure (HF) and healthy volunteers (HVs). Asterisks (*) represent individual outcomes and plusses (+) represent outlying measures.

In the evaluable population, within‐participant variability of *K* ^trans^ and *v*_*e*_ between MRI Sessions 1 and 2 for the HF and HV groups was generally small, demonstrating good repeatability for these measurements (Fig. 3a and b). Specifically, mean changes (95% CI) for the group difference between Sessions 1 and 2 mean total lung *K* ^trans^ were −0.10 minutes^−1^ (−0.40, 0.21) in the HF group and 0.19 minutes^−1^ (−0.10, 0.48) in the HV group (*P* = 0.52 and *P* = 0.18, coefficient of variation 32.4 and 41.3, respectively). The corresponding mean changes for *v*_*e*_ between MRI Sessions 1 and 2 were −0.12 (−0.27, 0.02) in the HF group and −0.05 (−0.19, 0.09) in the HV group (revealing a nonsignificant *P* = 0.09 and *P* = 0.46, respectively).

Comparison of within‐group differences between MRI scans before and after the exercise test (MRI Session 2 vs. Session 3) showed no differences for the evaluable HF or HV groups. The *K* ^trans^ mean changes (95% CI) were −0.02 minutes^−1^ (−0.07, 0.03) in the HF group and −0.05 minutes^−1^ (−0.09, 0.00) in the HV group (*P* = 0.43 and *P* = 0.06 respectively); the *v*_*e*_ mean changes (95% CI) were 0.02 (−0.03, 0.08) in the HF group and −0.01 (−0.06, 0.04) in the HV group (*P* = 0.42 and *P* = 0. 79, respectively) (Table 2). Details on the exercise approach are provided in the Supplementary Methods.

**TABLE 2:** Analysis of Total Lung *K* ^trans^ and *v*_*e*_ in the HF and HV Groups Before and After Exercise (Evaluable Population^a^)

Comparison of Interest	N	LS Mean (SE)		Mean Change^b^ (95% CI)	*P*‐Value
		Session 3	Session 2		
*K* ^trans^ (min^−1^)					
HV: Session 3 vs. Session 2	17	0.21 (0.02)	0.26 (0.02)	−0.05 (−0.09, 0.00)	0.06
HF: Session 3 vs. Session 2	12	0.18 (0.02)	0.20 (0.03)	−0.02 (−0.07, 0.03)	0.43
*v*_*e*_					
HV: Session 3 vs. Session 2	17	0.31 (0.03)	0.32 (0.03)	−0.01 (−0.06, 0.04)	0.79
HF: Session 3 vs. Session 2	12	0.46 (0.03)	0.44 (0.03)	0.02 (−0.03, 0.08)	0.42

CI = confidence interval; HF = heart failure; HV = healthy volunteers; *K* ^trans^ = exchange rate; LS = least squares; SE = standard error; *v*_*e*_ = interstitial volume fraction.

^a^ Evaluable population: All HF and HV participants included in the full population aged ≥40 years.

^b^ Mean changes for the change from MRI Session 2 for the comparison of postexercise in Session 3 vs. Session 2.

### Comparison of Imaging Parameters in Participants with HF vs. HV

**FIGURE 4:**
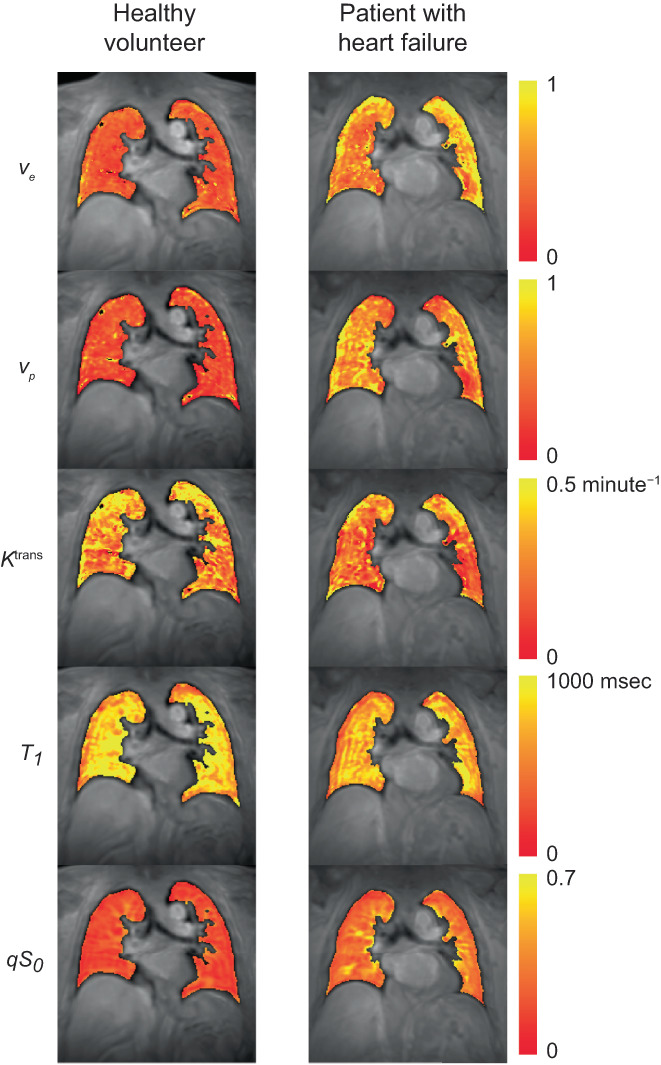
Example parameter maps for a healthy volunteer and a patient with heart failure. A single coronal slice of the 3D volume is shown in a similar anatomical location for both subjects.

**FIGURE 5:**
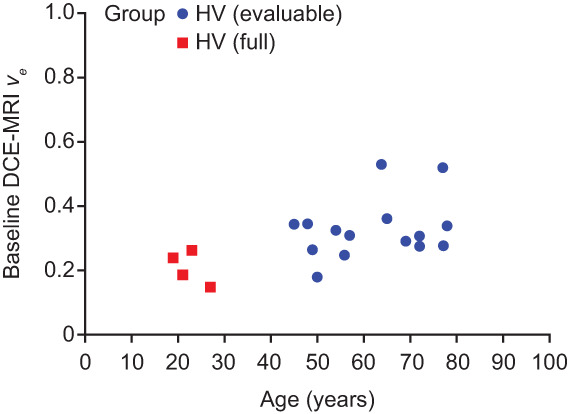
MRI Session 2 *v*_*e*_ for total lung vs. age in the HV group. Note that the data of two HV subjects were excluded due to bulk subject motion or severe cardiac motion artifact.

Plots of median total lung *K* ^trans^, *v*_*e*_, *T*_1_ relaxation time, *qS*_0_, *qS*_0_ *v*_*e*_ (post hoc), and mean *v*_*p*_ for the evaluable HF and HV groups across the three MRI sessions are shown in Fig. 3. Comparison of median lung *K* ^trans^ values showed no difference between the two groups at each visit (mean change for group difference [95% CI]: MRI Session 1: −0.01 minutes^−1^ [−0.10, 0.07], *P* = 0.74; MRI Session 2: −0.05 minutes^−1^ [−0.13, 0.02], *P* = 0.17; and MRI Session 3: −0.03 minutes^−1^ [−0.09, 0.03], *P* = 0.37; Fig. 3a). Median lung *v*_*e*_ was greater in participants with HF than HV at each session (mean change for group difference [95% CI]: MRI Session 1: 0.16 [0.06, 0.26], *P* = 0.003; MRI Session 2: 0.11 [0.02, 0.21], *P* = 0.02; and MRI Session 3: 0.14 [0.06, 0.23], *P* = 0.002; Figs 3b and 4). *K* ^trans^ and *v*_*e*_ observations in the total lung were consistent with findings for the four lung subregions assessed (apical left lung, basal left lung, apical right lung, and basal right lung; Table S1). Mean lung *v*_*p*_ did not show a difference between the HF and HV groups at each scan, and the median *v*_*p*_ (95% CI) for the HF group at Sessions 1, 2, and 3 was 0.52 (0.41. 0.66) vs. 0.42 (0.36, 0.53), 0.50 (0.42, 0.70) vs. 0.47 (0.36, 0.55), and 0.51 (0.44, 0.67) vs. 0.47 (0.40, 0.61) (Fig. 3f). There was also no difference in median total lung values for *T*_1_ relaxation time or *qS*_0_ between the HF and HV groups (Fig. 3c and d). Median *T*_1_ relaxation times (95% CI) for the HF group at Sessions 1, 2, and 3 were 729 (651, 818), 816 (666, 842), and 784 (679, 831), respectively; for the HV group, these values were 789 (756, 858), 792 (739, 840), and 724 (688, 786), respectively. Median *q*S_0_ (95% CI) for the HF group at Sessions 1, 2, and 3 was 0.27 (0.24, 0.29) AU, 0.26 (0.23, 0.30) AU, and 0.26 (0.23, 0.29) AU, respectively; for the HV group, these values were 0.24 (0.22, 0.26) AU, 0.27 (0.25, 0.29) AU, and 0.24 (0.22, 0.25) AU, respectively. Total lung *qS*_0_ *v*_*e*_ tended to be greater in participants with HF than HV at each visit (Fig. 3e). Median total lung *q*S_0_ *v*_*e*_ (95% CI) for the HF group vs. the HV group at Sessions 1, 2, and 3 was 0.14 (0.11, 0.16) vs. 0.07 (0.07, 0.11), 0.11 (0.09, 0.15) vs. 0.08 (0.08, 0.11), and 0.12 (0.10, 0.15) vs. 0.08 (0.07, 0.09), respectively. The correlation coefficient of *v*_*e*_ at baseline (MRI Session 2) with the age of HV participants revealed a moderate positive linear association (*r* = 0.55 [*P* = 0.01]) (Fig. 5). The *K* ^trans^, *v*_*e*_, *v*_*p*_, and *qS*_0_ *v*_*e*_ parameters were excluded post analysis from some HV for the following reasons: from three volunteers due to incorrect contrast agent dose at Session 1, from two volunteers due to bulk subject motion or severe cardiac motion artifact at Session 2, and from one volunteer due to severe cardiac motion artifact at Session 3. The *T*_1_ and *qS*_0_ parameters were also excluded from one of the aforementioned volunteers due to the severe cardiac artifact at Session 2. The *qS*_0_ was excluded from one volunteer due to a technical error at Session 2.

### Imaging Measurements in Participants with ADHF

**FIGURE 6:**
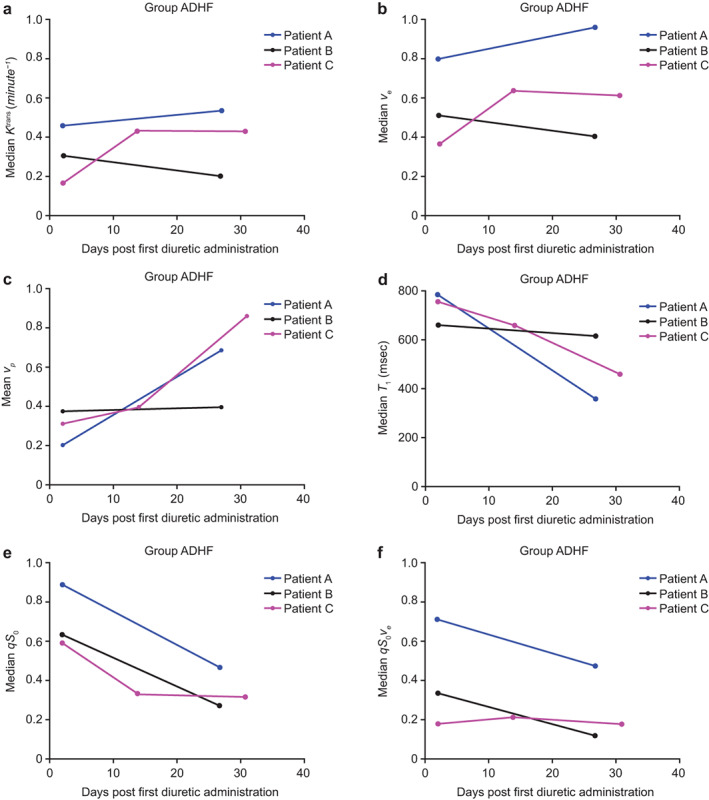
(**a**) *K* ^trans^, (**b**) *v*_*e*_, (**c**) *v*_*p*_, (**d**) *T*_1_ relaxation time, (**e**) *qS*_0_, and (**f**) *qS*_0_ *v*_*e*_ across visits for total lung in individual participants with acute decompensated heart failure (ADHF) (full population). Note that the data for patient C were considered substandard due to issues with contrast agent dynamics and possibly high lung fluid levels on Day 1, in addition to an incorrect acquisition protocol being used on the Day 14 rescan and motion during all scans.

The plots for total lung *K* ^trans^, *v*_*e*_, and *qS*_0_ for the three participants in the ADHF group before and after resolution of lung congestion (MRI Session 1 vs. Session 2/3) are shown in Fig. 6. There was no clear pattern of change in *K* ^trans^ or *v*_*e*_ across the visits in the ADHF group (Fig. 6a and b). However, mean *v*_*p*_ was higher in MRI Sessions 2 and 3 than Session 1 for all participants (Fig. 6c). In contrast, *T*_1_ relaxation time, *qS*_0_ and *qS*_0_ *v*_*e*_ were notably higher in MRI Session 1 than in MRI Session 2 or 3 for all three ADHF participants (Fig. 6d–f). Individual median *K* ^trans^, *v*_*e*_, *T*_1_, *qS*_0_, *qS*_0*ve*_, and mean *v*_*p*_ values derived in total lung are provided in Table S2.

### Procedural Tolerability

Study procedures were well tolerated with no serious adverse effects by participants in all three groups. There were no clinically significant findings reported from ECG or vital signs. As expected, all vital signs (systolic blood pressure, diastolic blood pressure, respiratory rate, and heart rate) were increased following the maximal exercise and constant workload tests compared with pre‐exercise values in HV and participants with HF.

## Discussion

We compared our findings in participants with HF with HV and found no significant between‐group differences in contrast agent transfer from the vascular to interstitial space (*K* ^trans^). This finding is in contrast to our prior assumption that interstitial engorgement during HF is in part due to alteration in the membrane between vascular and alveolar space. While it is possible that differences were present that were below the sensitivity of our chosen methods, the consistent *K* ^trans^ (and *v*_*e*_) 1‐week repeatability that we observed suggests that any difference is likely to be small. As we expected, *v*_*e*_ was significantly greater in participants with chronic HF than in HV, suggesting redistribution of fluid into the interstitial lung space. This observation was consistent across all three MRI sessions and for all four subregions of the lung assessed. There was no clear difference in mean lung *v*_*p*_ between the HF and HV groups, indicating that there is no evidence to support the possibility that plasma volume is the underlying basis for the *v*_*e*_ differential. Among these patients, *K* ^trans^ and *v*_*e*_ measurements were also repeatable with relatively small, nonstatistically significant within‐patient differences between MRI scans approximately 1 week apart. *v*_*e*_ in the ADHF cohort fell within the range observed for chronic HF; however, limited numbers (*n* = 3) prevented an appropriate comparison and further study would be needed to confirm this observation. The lack of change in *v*_*e*_ following intervention in the ADHF cohort suggests that *v*_*e*_ alone cannot fully describe changes in interstitial fluid. Further, a short transient period of exercise did not alter the *v*_*e*_ findings.

Lung water density enrichment in HF patients has been recognized previously by Thompson et al, 2019 using MR (spin echo pulse sequence, HASTE).^5^ As anticipated, these findings correlated with pulmonary artery pressures. We surmised that the high lung fluid volume is predominantly related to tissue water and not critically influenced by an increase in vascular volumes. Initial data by West and colleagues^17^ on permeability modification in HF, along with more recent data on transient receptor potential vanilloid (TRPV4) channels that implicated mechanosensitive pressure‐induced changes in the endothelium, led us to hypothesize that higher pulmonary pressures would change tissue permeability, amplify the shift in water to the lung tissue space, and thereby offer another target for HF therapeutics via a novel approach with TRPV4 antagonism. Therefore, the aim was to demonstrate whether we could assess permeability, as indicated by *K* ^trans^, as well as differentiate tissue fluid spaces, *v*_*e*_ and *v*_*p*_. This would need to be accomplished with comparatively small scan‐to‐scan variances to provide biomarkers that might have relevance for assessing interventions. This DCE‐MRI technology offers key advantages since it has the ability to distinguish the tissue compartments underpinning the differences in lung density seen in HF, an ability that is absent from conventional MRI methods and CT. One consideration was whether this approach was feasible in standard MRI scanners or whether upright scanning may be a preferred modality in this patient cohort. We opted for the standard MRI approach in view of its greater availability and considering what can be achieved in MR clinics; if specialized equipment or nonstandard pulse sequences had been used, it would be difficult to implement in large multicenter drug trials or in the clinic. In addition, previous work in the lung used half or full dose of contrast agent.^18^, ^19^ A half‐dose of contrast agent was used here to balance signal nonlinearity concerns with maintaining sufficient contrast‐to‐noise to measure the leakage phase of the contrast agent. Although simpler approaches for measuring lung congestion are available (such as ultrashort echo time) that can reflect overall tissue density, they are limited in use as they cannot distinguish between fluid compartments. The measurement of *v*_*e*_ in this study has been included to provide that specificity.

While the HF and TRPV4 literature would suggest that the stressor and physiologic impact of enhanced pressures on the vascular endothelium enhances permeability, our data in the lung did not provide evidence to support this mechanism. *K* ^trans^ was no different in HF patients than in HV. These findings, in the context of a higher *v*_*e*_, support the notion that an enhanced and maintained vascular pulmonary artery pressure is likely to be the main explanation for the comparatively increased tissue fluid content over the fraction of fluid in the vascular space. As West and colleagues have reported,^17^ chronic changes in the vascular membrane after time in the congested state allow for fluid shifts at lower pressures in patients with HF than in individuals without HF. This may therefore reflect not a permeability change, but rather a differential in threshold. To consider these hypotheses, we performed three tests in participants with HF. First, we nested an exercise element to determine whether perturbations in cardiovascular hemodynamics would alter fluid distribution in different compartments. A transient exercise component, ostensibly briefly raising pulmonary pressures, did not result in any changes in outcome parameters. This observation suggests that the elevation in pressures do not change the permeability or that transient pulmonary artery pressure rises are inadequate to modify tissue fluid. While this may represent an inadequate exercise stimulus, it may also indicate rapid resolution during the time it took to place the patient back into the MR machine and undergo DCE‐MRI, that being supine within the scanner negates the effect, or due to insufficient sensitivity of the technique. Second, we recruited a small number of participants with dramatic lung water congestion in ADHF and performed the same assessments. Baseline *v*_*e*_ values in these ADHF participants were not higher than those seen in the participants with HF. As *v*_*e*_ measurements are fractional volumes and given the overall increase in tissue water, we could surmise that the *v*_*p*_ or intracellular volumes were also increased, so that all lung tissues and vascular spaces were equally engorged (i.e., no distributional difference among the various MR‐apparent water locations in the lung). Finally, we retested the participants with ADHF after the provision of diuretics and realized a suggested pattern which recognizes an augmented vascular volume, while overall volume is decreasing. The physiology of resolution from acute congestion is unknown, and this represents one of the first detailed mechanistic phenotyping of this phenomenon using such a granular instrument. As patients generally feel better as their overall lung volumes decrease (i.e., less short of breath), it may suggest that interstitial volumes primarily signal central respiratory centers.

In summary, the cross‐sectional difference in *v*_*e*_ between the HF and controls is compelling; however, any dynamic changes due to exercise (or the acute HF setting) could not be elucidated. This fits with noted technical and biologic deficiencies but also remains consistent with potential fibrotic changes reported as inherent in long‐term HF patients.^17^ However, caution should be urged since this parameter could be confounded by the volume of lung fluid present as demonstrated in our ADHF cohort.

### Limitations

MR technical issues limit our conviction regarding the *v*_*e*_ and *K* ^trans^ measurements. *K* ^trans^ is a measure of gadolinium‐based contrast agent permeability and may not be fully reflective of permeability to fluid. DCE‐MRI alone cannot directly measure intracellular volume or fractional air volume, with estimates of *v*_*e*_ reflecting the proportion of MRI‐visible tissue occupied by the leakage space accessible to contrast agent, rather than a measurement of the leakage volume in mL. Therefore, changes in these parameters are not accounted for in the analysis. The use of estimates of lung tissue density via our *qS*_0_ measurements provides a mechanism for correcting for fractional air volume. Nevertheless, while *qS*_0_ provides a useful *estimate* of proton density, it is not an exact measure of proton density (although it is strongly proton‐density‐weighted). Furthermore, *qS*_0_ does not correct for differences in*T*_2_ and $T_{2}^{*}$ between lung parenchyma and muscle and does not correct for the spatial variation in coil sensitivity within each imaging slice, meaning that the current methods are susceptible to the placement of the muscle region of interest. Further refinement of the method is likely to improve accuracy of the tissue density estimates available, and therefore, the likely accuracy of *v*_*e*_ modulated by *qS*_0_, to provide the absolute estimates of the lung fluid space accessible to contrast agent. In addition, fibrosis can interfere with fluid metrics, and as such fibrosis enrichment reported in HF patients may be explained by the higher *v*_*e*_ in these HF individuals.

While the primary outcome variables are automatically produced via machine algorithm, we did not explicitly measure intrarater repeatability of the stages of the analysis that required manual intervention, including the definition of the arterial input function and the lung and muscle segmentation steps. However, the measurements were performed under blinded conditions, and any impact of poorly repeatable manual intervention can be inferred to be small, given the good repeatability for the key parameters extracted from the tissues of interest.

There are further clinical issues to consider. Exercise may lead to fluid changes in non‐MRI‐visible compartments. Additionally, a “true” nonchallenge baseline was not available, as patients who were not exercising spent 30 minutes in a supine position before the two baseline scans, an activity that is likely to have been a passive challenge for HF patients. There were only three participants with ADHF and further study in a larger ADHF cohort is required.

Finally, the study was conducted in a relatively small population from a single center in the United Kingdom; therefore, further studies in larger, more diverse populations will be required, in conjunction with other methods for fluid assessment. One possibility is the use of ultrashort echo time imaging, which would provide a measure of overall tissue density, but would not distinguish fluid build‐up. Despite the relatively small participant numbers, we demonstrate in this study that DCE‐MRI can be employed safely and is well tolerated in chronic stable as well as in the decompensated HF setting to assess lung edema despite supine scanning in moderately unwell patients. However, the utility and applicability of supine MRI scanning in the emergency room as a diagnostic technique remains limited. Despite this limitation, there may be opportunities to assess response to drug therapy and in the development of novel therapeutic agents for HF in a less compromised patient setting.

## Conclusion

In conclusion, our study suggests that DCE‐MRI could be used to identify and characterize increased interstitial lung fluid, as measured by *v*_*e*_ in the context of chronic HF. Physiologic findings from this study suggest lack of a permeability change in participants with HF compared with HV, and the enrichment of vascular water upon the progressive resolution of congestion in the context of diuretic‐treated ADHF. This may have implications for allowing early identification of patients at greatest clinical risk, HF treatment paradigms, and novel HF therapy development to maximize patient outcomes.

## Conflict of Interest

J.C. was the Principal Investigator for this study. He is employed by Cambridge University Hospitals NHS Foundation Trust but is obligated via a secondment agreement between the Trust and GSK to spend 50% of his NHS time on GSK clinical research such as this project. He receives no employee benefits from GSK. A.R. was an employee of Bioxydyn Ltd and, subsequently, an employee of GSK, at the time of the study. A.R. was a consultant to GSK during manuscript preparation. C.R. owns stock in Bioxydyn Ltd. R.A.S. has received lecturer honoraria from Bayer and GE. G.J.M.P. is a shareholder and director of Bioxydyn Ltd. D.S. was an employee of GSK at the time of the study and is now President of BioView Consulting, LLC. L.S.‐B., D.F., S.K., and R.S. are employees of GSK and hold stocks/shares in the company. R.L.J. was an employee of GSK at the time of the study. J.M. was a PhD student at the University of Cambridge (at the Division of Experimental Medicine and Immunotherapeutics) at the time of study and now works at AstraZeneca, Cambridge, UK. S.L. is a company director of Amallis Consulting and reports fees from GSK and GE Healthcare. C.M., I.P., and M.J.G. report no disclosures.

## Supporting information


**Appendix S1**: Supporting information

## Data Availability

Anonymized individual participant data and study documents can be requested for further research from www.clinicalstudydatarequest.com.

## References

[1] Gheorghiade M , Follath F , Ponikowski P , et al. Assessing and grading congestion in acute heart failure: A scientific statement from the acute heart failure committee of the heart failure association of the European Society of Cardiology and endorsed by the European Society of Intensive Care Medicine. Eur J Heart Fail 2010;12:423‐433. 20354029 10.1093/eurjhf/hfq045

[2] Assaad S , Kratzert WB , Shelley B , Friedman MB , Perrino A Jr . Assessment of pulmonary edema: Principles and practice. J Cardiothorac Vasc Anesth 2018;32:901‐914. 29174750 10.1053/j.jvca.2017.08.028

[3] Jozwiak M , Teboul J‐L , Monnet X . Extravascular lung water in critical care: Recent advances and clinical applications. Ann Intensive Care 2015;5:38. 26546321 10.1186/s13613-015-0081-9 PMC4636545

[4] Chow K , Toma M , Esch B , et al. Comparison of MRI‐derived pulmonary edema measures with LVEDP and serum BNP. J Cardiovasc Magn Reson 2009;11:P41.

[5] Thompson RB , Chow K , Pagano JJ , et al. Quantification of lung water in heart failure using cardiovascular magnetic resonance imaging. J Cardiovasc Magn Reson 2019;21:58. 31511018 10.1186/s12968-019-0567-y PMC6739968

[6] Sourbron SP , Buckley DL . On the scope and interpretation of the Tofts models for DCE‐MRI. Magn Reson Med 2011;66:735‐745. 21384424 10.1002/mrm.22861

[7] Murray JF . Pulmonary edema: Pathophysiology and diagnosis. Int J Tuberc Lung Dis 2011;15:155‐160. 21219673

[8] Melenovsky V , Andersen MJ , Andress K , Reddy YN , Borlaug BA . Lung congestion in chronic heart failure: Haemodynamic, clinical, and prognostic implications. Eur J Heart Fail 2015;17:1161‐1171. 26467180 10.1002/ejhf.417

[9] Naish JH , McGrath DM , Hutchinson CE . Increased pulmonary capillary permeability in smokers as measured by DCE‐MRI. In: *Proceedings of the International Society for Magnetic Resonance*, Vol. 16; 2008, p 401.

[10] Chang YC , Yu CJ , Chen CM , et al. Dynamic contrast‐enhanced MRI in advanced nonsmall‐cell lung cancer patients treated with first‐line bevacizumab, gemcitabine, and cisplatin. J Magn Reson Imaging 2012;36:387‐396. 22517425 10.1002/jmri.23660

[11] Naish JH , Kershaw LE , Buckley DL , Jackson A , Waterton JC , Parker GJ . Modeling of contrast agent kinetics in the lung using T1‐weighted dynamic contrast‐enhanced MRI. Magn Reson Med 2009;61:1507‐1514. 19319896 10.1002/mrm.21814

[12] Saporito S , Herold IH , Houthuizen P , et al. Model‐based characterization of the transpulmonary circulation by dynamic contrast‐enhanced magnetic resonance imaging in heart failure and healthy volunteers. Invest Radiol 2016;51:720‐727. 27379699 10.1097/RLI.0000000000000304

[13] New York Heart Association . The criteria Committee of the New York Heart Association. Nomenclature and criteria for diagnosis of diseases of the heart and great vessels. 9th ed. Boston: Little, Brown & Co; 1994. p 253‐256.

[14] Fram EK , Herfkens RJ , Johnson GA , et al. Rapid calculation of T1 using variable flip angle gradient refocused imaging. J Magn Reson Imaging 1987;5:201‐208. 10.1016/0730-725x(87)90021-x 3626789

[15] Tofts PS , Brix G , Buckley DL , et al. Estimating kinetic parameters from dynamic contrast‐enhanced T(1)‐weighted MRI of a diffusable tracer: Standardized quantities and symbols. J Magn Reson Imaging 1999;10:223‐232. 10508281 10.1002/(sici)1522-2586(199909)10:3<223::aid-jmri2>3.0.co;2-s

[16] Zhang WJ , Hubbard Cristinacce PL , Bondesson E , et al. MR quantitative equilibrium signal mapping: A reliable alternative to CT in the assessment of emphysema in patients with chronic obstructive pulmonary disease. Radiology 2015;275:579‐588. 25575114 10.1148/radiol.14132953

[17] West JB , Mathieu‐Costello O . Vulnerability of pulmonary capillaries in heart disease. Circulation 1995;92:622‐631. 7634477 10.1161/01.cir.92.3.622

[18] Ingrisch M , Maxien D , Schwab F , Reiser MF , Nikolaou K , Dietrich O . Assessment of pulmonary perfusion with breath‐hold and free‐breathing dynamic contrast‐enhanced magnetic resonance imaging: Quantification and reproducibility. Invest Radiol 2014;49:382‐389. 24473368 10.1097/RLI.0000000000000020

[19] Ley‐Zaporozhan J , Molinari F , Risse F , et al. Repeatability and reproducibility of quantitative whole‐lung perfusion magnetic resonance imaging. J Thorac Imaging 2011;26:230‐239. 20818278 10.1097/RTI.0b013e3181e48c36

