# Supplementary material for: Evaluation of Dynamic Contrast‐Enhanced MRI Measures of Lung Congestion and Endothelial Permeability in Heart Failure: A Prospective Method Validation Study
Source: J Magn Reson Imaging. 2022 Mar 27;56(2):450–61. doi: 10.1002/jmri.28174 (PMC9544235; doi:10.1002/jmri.28174)
Supplement: Supplementary file 1 — Appendix S1: Supporting information [file JMRI-56-450-s001.docx]

**Supplementary Information for:**

**Evaluation of Dynamic Contrast-Enhanced MRI Measures of Lung Congestion and Endothelial Permeability in Heart Failure: A Prospective Method Validation Study**

# Supplementary Methods

## Study design

Exercise testing included two exercise challenges over 2 days on a semi-recumbant cycle ergometer; a ramped cardiopulmonary exercise testing (CPET) protocol to exhaustion to establish the maximum oxygen consumption for each individual, and a constant workload exercise protocol, conducted one day later (1). The rationale for this mode of exercise testing was based on previous observations of decreased conductance across the capillary-alveolar barrier consistent with pulmonary edema, following a period of high intensity constant workload exercise in patients with heart failure (2). For the maximal exercise test, baseline assessments were performed while patients were seated on the cycle ergometer for approximately 3 minutes. They were then asked to commence cycling at a workload of 20 Watts, at a pedaling rate of 50 revolutions per minute (rpm) for two minutes. The workload was subsequently increased by 20 Watts every two minutes until volitional fatigue. Breath-by-breath gas measurements and heart rate were recorded throughout exercise. At the end of each workload, Borg Rating of Perceived Exertion (RPE) and blood pressure were obtained. At peak exercise, heart rate, blood pressure, Borg RPE and breath-by-breath gas exchange measurements were recorded. Respiratory rate at the end of exercise was also recorded. For the constant workload test, patients were asked to perform cycling exercise for 10 minutes at a workload corresponding to 75% of the peak workload achieved during the maximal CPET protocol. Pedaling rate was again held constant at 50 rpm. Breath-by-breath gas measurements and heart rate were again measured throughout the exercise period. Blood pressure, respiratory rate, and Borg Rating of Perceived Exertion (RPE) were also obtained at the completion of the exercise period.

Lung congestion in participants with acute decompensated heart failure (ADHF) was deemed to be resolved if judged so by the investigator, using lung auscultation and/or chest x-ray. Patients whose lung congestion had not resolved by Session 2 underwent a delayed second MRI scan at an extra session.

## Assessments

During MRI scanning, participants were placed in the supine position in the scanner during free breathing and prepared for intravenous contrast agent administration. Participants were re-positioned if image artefacts (e.g., wrap-around) were observed prior to contrast agent administration. In the ADHF group, all participants received supplemental medical oxygen during imaging exams via Venturi oxygen mask or nasal prongs, beginning at least 15 minutes before entering the scanner and matched between scans to avoid confounding. Participants were monitored using a pulse oximeter throughout each scan.

Healthy volunteers (HV) and participants with heart failure (HF) underwent a maximal exercise test to exhaustion or dyspnea to determine their individual maximal cardiovascular thresholds on the first exercise session. Subsequent to this and on a separate day just prior to the third MRI imaging session, participants underwent a constant workload exercise test for 10 minutes at 75–80% of the peak work rate achieved during the prior maximal exercise test.

Intravenous contrast agent was injected at the 15^th^ volume (0.05 ml/kg; half-dose gadolinium-based contrast agent [Gadovist, Bayer, Berkshire, UK] administered by power injector at 1.5 mL/s, followed by a 25 mL saline flush). Total scan time was approximately 10 mins, allowing for inclusion of participants with mild orthopnea.

The MRI equilibrium signal in the lung, *qS_0_*, was extracted from the variable flip angle *T_1_* mapping process, normalized to signal in skeletal muscle and calculated for all participants (3). The product of *qS_0_* and interstitial volume fraction [*v_e_*] (referred to as *qS_0_v_e_* hereafter) was calculated *post hoc* to correct *v_e_* for non-MR-visible compartments (e.g., air) by normalizing it to *qS_0_*.

Where wrap-around artefact was discovered (resulting in non-analyzable regions of the lung), a modified imaging protocol was developed and used in participants with large abdominal and chest cavities (as evaluated by the radiographers) at each imaging session. This protocol included greater antero-posterior coverage by increasing the slice thickness and number of slices, while reducing the phase matrix to achieve a temporal resolution comparable to the standard protocol.

In Session 3, dyspnea score and respiratory rate were recorded before and after the exercise tests (maximal exercise test and constant workload test [10-minute cycle exercise at 75–80% of the peak work rate achieved during the maximal exercise test]).

Vital signs including heart rate, respiratory rate, systolic blood pressure, and diastolic blood pressure were monitored throughout the MRI. Heart rate and blood pressure measurements were collected with the participant in the semi-supine position, after they had rested for 5 minutes; for HV and HF groups, additional measurements of heart rate and blood pressure were made during exercise testing. Respiratory rate was recorded over a 60-second period; for HV and HF groups, respiratory rate was measured after the MRI scan in Sessions 1 and 2. In Session 3, respiratory rate was measured immediately before and after exercise tests, and after MRI scanning. For the ADHF group, respiratory rate was measured at screening and prior to the MRI scan in all three sessions if required. For HV and HF groups, 12-lead electrocardiograms (ECGs) were obtained using an ECG machine that automatically calculated the heart rate and measured PR, QRS, QT, and QTc intervals; ECGs were obtained with the participant in the semi-supine position, after they had rested for 5 minutes. Urinalysis was conducted for HV and HF groups; the specific gravity was measured; pH, glucose, protein, blood and ketone were assessed by dipstick; and microscopic examination was conducted if blood or protein was abnormal. The investigator and/or site staff were responsible for detecting, documenting, and reporting of any adverse events (AEs) or serious AEs (SAEs). AEs determined to be related to study participation were recorded from the time the consent to participate in the study was obtained until the last visit.

## Endpoints

Additional exploratory endpoints for the HF and HV groups included *v_p_*, *T_1_* relaxation time, and the *qS*_0_ extracted from the MRI variable flip angle *T_1_* mapping process normalized to signal in skeletal muscle (3). Change in *v_p_*, *T_1_* relaxation time and *qS*_0_ from baseline to resolution of lung congestion was also assessed in the ADHF group. A *post hoc* analysis of *qS*_0_*v_e_* in the HF, HV and ADHF groups, and baseline *v_e_* by participant age in the HF and HV groups was also performed.

## Data analyses

MRI data were quality controlled and corrected for breathing motion using a non-linear image registration algorithm (4). The lungs were segmented from the thorax using a semi-automatic thresholding technique, with the major vasculature in the lung removed and any artefacts due to partial-voluming effects at edges of lungs omitted. Any major artefacts of cardiac motion were also removed from the lung segmentation. The three timepoints, for each participant, were segmented together in one session to ensure consistency across timepoints. Segmentation was performed by Bioxydyn, with each lung segmentation quality controlled by the second operator to ensure correctness of segmentation and consistency across timepoints. The result was a complete 3D segmentation of the lungs for each participant at each timepoint. A correction was applied to the variable flip angle data to correct for radiofrequency transmission non-uniformly using data from phantom acquisitions as well as the *T_1_* values in blood. *T_1_* relaxation time was estimated voxel-by-voxel by fitting the standard spoiled gradient echo signal model to the VFA data, additionally providing an estimate of the relative equilibrium signal (*S_0_*) (5). *qS*_0_ was calculated voxel-by-voxel within each slice by normalizing the lung *S_0_* to the *S_0_* measured from a small, manually defined, skeletal muscle region of interest (ROI) within the slice. Muscle ROIs were drawn manually by Bioxydyn. A small circular ROI in each slice containing lung tissue was drawn either in the neck or shoulder muscle, depending on the participant composition, positioning in the scanner, and field of view (FOV) placement. This approach ensured signal homogeneity in the tissue selected across slices. Repeated visits were analyzed together to ensure consistency in the ROI placement between timepoints. Muscle ROIs were quality-controlled by the second operator to ensure correctness of segmentation and consistency across timepoints. DCE-MRI analysis was then performed by pharmacokinetic modeling using the extended Tofts model (6) to quantify *v_e_*, *K*^trans^ and *v_p_* on a voxel-wise basis. Where possible, an individually measured arterial input function (AIF) was measured by manually defining a region of interest in the pulmonary artery. The ROIs for AIF definition were created by Bioxydyn and confirmed by a second operator both for correctness of placement in the pulmonary artery as well as acceptability of the AIF shape for modeling (e.g., minimal acceptable noise in curve). In cases where individually measured extraction of an AIF was not possible (for example, in cases with significant bulk motion of the pulmonary artery or partial volume effect in the pulmonary artery) due to slice position, a population AIF (created from the baseline visit AIF of a subset of the participants) was used. AIFs were corrected for blood hematocrit using patient-specific hematocrit values measured at each session.

# Supplementary Results

## Supplementary Table 1: Point estimates for the difference between the HF and HV groups in total and regional lung *K*^trans^ and *v_e_* (Evaluable Population)

|  | **MRI Session 1** | | **MRI Session 2** | | **MRI Session 3** | |
| --- | --- | --- | --- | --- | --- | --- |
|  | **Point estimate^a^ (95% CI)** | **p-value** | **Point estimate^a^ (95% CI)** | **p-value** | **Point estimate^a^ (95% CI)** | **p-value** |
| ***K*^trans^ (min^–1^)** | | | | | | |
| **Total lung** | –0.01 (–0.10, 0.07) | 0.738 | –0.05 (–0.13, 0.02) | 0.173 | –0.03 (–0.09, 0.03) | 0.368 |
| Upper left lung | 0.00 (–0.07, 0.07) | 0.100 | –0.06 (–0.14, 0.02) | 0.131 | –0.02 (–0.07, 0.04) | 0.547 |
| Lower left lung | 0.04 (–0.04, 0.12) | 0.291 | –0.01 (–0.09, 0.07) | 0.843 | –0.01 (–0.07, 0.05) | 0.679 |
| Upper right lung | –0.04 (–0.14, 0.06) | 0.459 | –0.05 (–0.13, 0.03) | 0.185 | –0.02 (–0.10, 0.05) | 0.481 |
| Lower right lung | –0.01 (–0.10, 0.08) | 0.794 | –0.04 (–0.13, 0.05) | 0.341 | –0.00 (–0.08, 0.07) | 0.938 |
| ***v_e_*** | | | | | | |
| **Total lung** | 0.16 (0.06, 0.26) | 0.003 | 0.11 (0.02, 0.21) | 0.016 | 0.14 (0.06, 0.23) | 0.002 |
| Upper left lung | 0.18 (0.08, 0.27) | <0.001 | 0.11 (0.02, 0.21) | 0.016 | 0.15 (0.08, 0.22) | <0.001 |
| Lower left lung | 0.20 (0.09, 0.31) | 0.001 | 0.18 (0.09, 0.26) | <0.001 | 0.21 (0.13, 0.30) | <0.001 |
| Upper right lung | 0.14 (0.03, 0.20) | 0.016 | 0.10 (–0.01, 0.20) | 0.066 | 0.11 (0.02, 0.21) | 0.025 |
| Lower right lung | 0.17 (0.05, 0.30) | 0.006 | 0.12 (0.03, 0.21) | 0.015 | 0.15 (0.05, 0.24) | 0.005 |

^a^Point estimates for the mean difference between the HF and HV groups at each session.

CI, confidence interval; HF, heart failure; HV, healthy volunteers; *K*^trans^, transfer constant; *v_e_*, interstitial volume fraction

## Supplementary Table 2. Imaging parameters with whole lung as the region of interest for individual participants with ADHF

| **Patient** | **Visit** | **Median *K*^trans^** | **Median *v_e_*** | **Mean *v_p_*** | **Median T_1_** | **Median *qS_0_*** | **Median *qS_0_v_e_*** | **Median days after diuretic** |
| --- | --- | --- | --- | --- | --- | --- | --- | --- |
| A | 1 | 0.4524 | 0.7940 | 0.2040 | 784.91 | 0.8875 | 0.7074 | 2 |
| A | 2 | 0.5306 | 0.9577 | 0.6880 | 365.71 | 0.4637 | 0.4678 | 27 |
| B | 1 | 0.3018 | 0.5086 | 0.3763 | 660.60 | 0.6334 | 0.3316 | 2 |
| B | 2 | 0.1952 | 0.4018 | 0.3965 | 619.50 | 0.2690 | 0.1141 | 27 |
| C | 1 | 0.1602 | 0.3540 | 0.3131 | 755.83 | 0.5898 | 0.1780 | 2 |
| C | 2 | 0.4304 | 0.6356 | 0.3962 | 661.71 | 0.3284 | 0.2081 | 14 |
| C | 3 | 0.4263 | 0.6083 | 0.8599 | 464.90 | 0.3159 | 0.1732 | 31 |

## Supplementary Figure 1. Scanning protocol


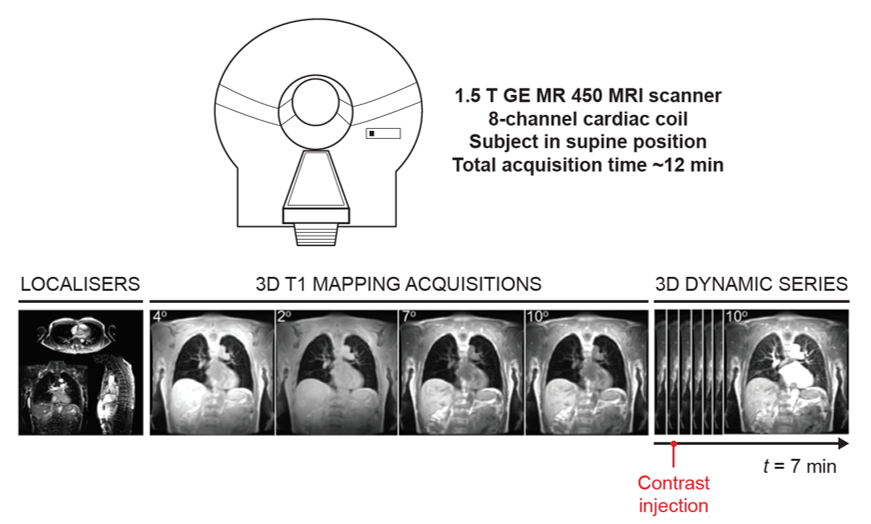


**References**

1. Agostoni P, Cattadori G, Bianchi M, Wasserman K. Exercise-induced pulmonary edema in heart failure. Circulation 2003;108:2666-2671.

2. Olson LJ, Snyder EM, Beck KC, Johnson BD. Reduced rate of alveolar-capillary recruitment and fall of pulmonary diffusing capacity during exercise in patients with heart failure. J Card Fail 2006;12:299-306.

3. Zhang WJ, Hubbard Cristinacce PL, Bondesson E, et al. MR Quantitative Equilibrium Signal Mapping: A Reliable Alternative to CT in the Assessment of Emphysema in Patients with Chronic Obstructive Pulmonary Disease. Radiology 2015;275:579–588.

4. Ley-Zaporozhan J, Molinari F, Risse F, et al. Repeatability and reproducibility of quantitative whole-lung perfusion magnetic resonance imaging. J Thorac Imaging 2011;26:230-239.

5. Fram EK, Herfkens RJ, Johnson GA, et al. Rapid calculation of T1 using variable flip angle gradient refocused imaging. JMRI 1987;5:201–208.

6. Tofts PS, Brix G, Buckley DL, et al. Estimating kinetic parameters from dynamic contrast-enhanced T(1)-weighted MRI of a diffusable tracer: standardized quantities and symbols. JMRI 1999;10:223–232.
